# Neutrophil-to-lymphocyte and platelet-to-lymphocyte ratios predict chemotherapy outcomes and prognosis in patients with colorectal cancer and synchronous liver metastasis

**DOI:** 10.1186/s12957-016-1044-9

**Published:** 2016-11-16

**Authors:** Yuchen Wu, Cong Li, Jiang Zhao, Li Yang, Fangqi Liu, Hongtu Zheng, Zhimin Wang, Ye Xu

**Affiliations:** 1 Department of Oncology, Shanghai Medical College, Shanghai, 200032 China; 2 Department of Colorectal Surgery, Fudan University Shanghai Cancer Center, Shanghai, 200032 China; 3 Department of Genetics, Shanghai-MOST Key Laboratory of Health and Disease Genomics, Chinese National Human Genome Center and Shanghai Industrial Technology Institute (SITI), Shanghai, 201203 China

**Keywords:** PLR, NLR, Synchronous colorectal liver metastasis, Prognosis, Chemotherapy response

## Abstract

**Background:**

Recent evidence indicates that inflammatory parameters could be useful to predict metastasis from colorectal cancer. However, their roles in predicting chemotherapy response and prognosis in patients with synchronous colorectal liver metastasis (CLM) are unknown.

**Methods:**

The clinical data and baseline laboratory parameters of 55 patients with synchronous CLM were retrospectively reviewed. All patients underwent palliative resection of the primary tumor and oxaliplatin-based chemotherapy. Two indices of systemic inflammation were reviewed—neutrophil-to-lymphocyte ratio (NLR) and platelet-to-lymphocyte ratio (PLR)—preoperatively and before the second cycle of chemotherapy. Associations between prognostic variables and tumor response, progression, and survival were investigated.

**Results:**

NLR < 4 and PLR < 150 were correlated with better disease control (*p* = 0.024 and 0.026, respectively). In univariate analysis, elevated NLR and PLR were significant prognostic factors for poor overall survival (OS) and progression-free survival (PFS). In multivariate analysis, PLR (*p* = 0.027), age (*p* = 0.018), resection of liver metastases (*p* = 0.017), and lactate dehydrogenase level (*p* = 0.011) were independent predictors of PFS, while resection of liver metastases was the only independent predictor of OS (*p* = 0.002). In addition, when patients were divided into groups according to changes in NLR and/or PLR, reduced NLR and PLR were associated with improved disease control (*p* = 0.038 and 0.025, respectively). Normalization of NLR also was associated with improved PFS.

**Conclusions:**

NLR and PLR are potentially useful clinical biomarkers to predict chemotherapy response in patients with synchronous CLM. PLR also may be useful to predict PFS in these patients.

**Electronic supplementary material:**

The online version of this article (doi:10.1186/s12957-016-1044-9) contains supplementary material, which is available to authorized users.

## Background

Colorectal cancer (CRC) is the third most common cancer worldwide and the fifth most common in China [1]. It is also the fifth most common cause of cancer-related death. Although there have been remarkable improvements in the treatment and management of CRC, outcomes remain poor, with approximately 40% of patients who undergo curative surgery dying from their disease [2], especially those with distant metastases.

In patients with synchronous colorectal liver metastasis (CLM), radical resection is the only curative therapy [3], which can increase 5-year survival up to 71% [4]. However, in unresectable or potentially resectable CLM, chemotherapy is a paramount consideration. Studies have shown that chemotherapy regimens can downstage 15 to 50% of patients from unresectable to resectable cancer [5]. However, there are numerous patients who cannot benefit from adjuvant chemotherapy, and identifying and developing biomarkers able to distinguish such patients is important.

Fortunately, several parameters for predicting survival in patients with CRC have been identified, including such inflammatory-based prognostic parameters as neutrophil-to-lymphocyte ratio (NLR), platelet-to-lymphocyte ratio (PLR), white blood cell count, and platelet count [6–9]. NLR, calculated as neutrophil count divided by lymphocyte count, is the most frequently reported marker and is involved in almost every stage of CRC [6, 10–12]. Increased NLR is associated with worse outcomes and insensitive response to adjuvant chemotherapy or radiotherapy [13, 14]. PLR, calculated as platelet count divided by lymphocyte count, is also gaining attention in some research [8].

It is unclear whether both indices are associated with chemotherapy response in patients with synchronous CLM treated with oxaliplatin-based chemotherapy after resection of primary lesions. Therefore, in this study, we examined the correlations of NLR and PLR with chemotherapy sensitivity and prognosis in these patients.

## Methods

### Patient selection, treatment, and follow-up

We retrospectively reviewed a database of patients treated in the Department of Colorectal Surgery at the Shanghai Cancer Center from June 2008 to December 2013. Patients who met the following criteria were selected: (a) initially diagnosed as having synchronous CLM; (b) evaluated as having potentially resectable cancer before any treatment; (c) Eastern Cooperative Oncology Group (ECOG) status <2; (d) available and complete clinical records, including pathologic diagnosis, treatment strategy, follow-up information, and laboratory data; and (e) treated with first-line chemotherapy. The exclusion criteria included patients who had either malignant tumors in other organs or systematic inflammatory or hematologic disease. Patients with complications from the primary tumor, such as obstruction or hemorrhage, also were excluded.

A total of 55 patients were finally included in the study. All patients underwent R0 resection of the primary tumor followed by oxaliplatin-based chemotherapy, including mFOLFOX6 (folic acid/fluorouracil plus oxaliplatin) or XELOX (capecitabine plus oxaliplatin). The duration from surgery to chemotherapy was within 1 month.

Laboratory tests and imaging examinations were performed every 3 months. The Response Evaluation Criteria in Solid Tumors was applied for evaluation of disease status. Radical hepatectomy of metastases was conducted if the patient had reached complete response (CR) or partial response (PR). Four patients who had reached stable disease (SD) after chemotherapy also underwent radical hepatectomy, as their metastases had contracted but they did not meet the baseline criteria of PR.

### Parameters evaluated

The following parameters were evaluated: sex, age, number of liver metastases, CLM resection, and chemotherapy protocol. Several pathologic parameters, including size, pathologic subtype, differentiation, location, tumor invasion, nodal status, lymphovascular invasion, perineural invasion, extranodal tumor deposits, and microsatellite instability, also were evaluated. Laboratory parameters, including NLR, PLR, alkaline phosphatase (ALP) level, lactic dehydrogenase (LDH) level, carcinoembryonic antigen level, and CA199 level, also were assessed.

The number of liver metastases was categorized according to the criteria between minor and major lesions [15]. Regarding pathologic diagnosis, expression of MLH1, MSH2, MLH6, and PMS2 was routinely evaluated. Any deficiency was considered as deficient mismatch repair. We used the X-tile Software (http://medicine.yale.edu/lab/rimm/research/) to determine the cutoff values of NLR and PLR based on the minimum *p* values from the log-rank chi-square statistics (Additional file 1). Finally, the cutoff values of NLR and PLR were set at 4 and 150, respectively, which were also in accordance with previous researches [6, 16, 17]. All parameters were evaluated preoperatively and before the second cycle of chemotherapy. Other parameters were presented according to the normal value range at our hospital.

### Statistical analysis

Progression-free survival (PFS) was defined as the duration from primary tumor resection to disease progression, while overall survival (OS) was defined as the duration from surgery to the date of death or the last date of follow-up.

All statistical analyses were performed by using SPSS version 20.0 (IBM Corporation, Armonk, NY, USA). Fisher’s exact test was used to compare chemotherapy response among groups. Survival curves were plotted by using the Kaplan-Meier method and analyzed by using the log-rank test. Univariate and multivariate analyses to identify prognostic predictors were performed by using Cox proportional hazard models. A *p* value of <0.05 was considered as significant in all analyses.

## Results

### Patient characteristics

All of the patients’ clinicopathologic features are summarized in Table 1. Patients were predominantly male (64%), and the median age was 59 years. Almost half of the patients had more than three liver metastases. Regarding the chemotherapy regimen, 21 patients (38%) received mFOLFOX6, while 34 (62%) received XELOX. Of these patients, 12 (22%) underwent radical resection of liver metastases.

**Table 1:** Characteristic variable

Characteristics	Category	No. of patients (%)
Clinical background		
Sex	Male	35 (64)
	Female	20 (36)
Age (years)	<60	28 (51)
	≥60	27 (49)
Number of liver metastasis	≤3	27 (49)
	>3	28 (51)
Liver metastasis resection	No	43 (78)
	Yes	12 (22)
First-line chemotherapy	FOLFOX	21 (38)
	XELOX	34 (62)
Surgical pathology		
Maximum size (cm)	≤4	29 (53)
	>4	26 (47)
Pathology	Adenocarcinoma	50 (91)
	Mucinous or signet-ring carcinoma	5 (9)
Differentiation	G1–G2	33 (60)
	G3–G4	22 (40)
Location	Colon	25 (45)
	Rectum	30 (55)
Tumor stage (T)	2	1 (2)
	3	13 (24)
	4	41 (74)
Nodal status (N)	0	11 (20)
	1	19 (35)
	2	25 (45)
Lymphovascular invasion	No	25 (45)
	Yes	30 (55)
Perineural invasion	No	28 (51)
	Yes	27 (49)
Extranodal tumor deposits	No	30 (55)
	Yes	25 (45)
MSI status	dMMR	4 (7)
	pMMR	51 (93)
Blood biochemical test		
NLR	<4	41 (75)
	≥4	14 (25)
PLR	<150	31 (56)
	≥150	24 (44)
ALP	Normal	46 (84)
	Elevated	9 (16)
LDH	Normal	44 (80)
	Elevated	11 (20)
Serum CA199 level	Normal	20 (36)
	Elevated	35 (64)
Serum CEA level	Normal	12 (22)
	Elevated	43 (78)

According to the pathologic results, the median maximum size of the primary lesion was 4 cm, and almost all of the lesions (91%) were identified as adenocarcinoma. Twenty-five patients (45%) suffered from colon cancer, while 30 (55%) suffered from rectal cancer. Most of the lesions (98%) reached an invasive depth of T3 to T4 with local nodal metastasis (80%). Furthermore, lymphovascular invasion, perineural invasion, and extranodal tumor deposits were almost evenly distributed, and most of the patients were proficient mismatch repair.

With regard to laboratory data, 41 patients (75%) had a low NLR, and 31 (56%) had a low PLR. Other biochemical parameters, such as ALP and LDH levels, were normal in most of the patients. On the contrary, most of the patients had elevated serum carcinoembryonic antigen and CA199 levels.

### NLR and PLR with regard to chemotherapy response

Objective chemotherapy response, including CR and PR, did not differ significantly between groups divided by preoperative NLR and PLR (*p* = 0.051 and 0.195, respectively; Table 2). However, disease control, including CR, PR, and SD, was significantly better in groups with comparatively lower preoperative NLR (56–21%, *p* = 0.024) and PLR (61–29%, *p* = 0.026) (Table 2).

**Table 2:** Chemotherapy response according to NLR and PLR before operation

Response	NLR			PLR		
	<4 (*N* = 41)	≥4 (*N* = 14)	*p* value	<150 (*N* = 31)	≥150 (*N* = 24)	*p* value
Objective response rate (CR + PR), % (cases)	20% (8)	0 (0)	0.051	19% (6)	8% (2)	0.195
Disease control rate (CR + PR + SD), % (cases)	56% (23)	21% (3)	0.024*	61% (19)	29% (7)	0.026*

*A *p* value ≤0.05 was considered statistically significant

### Prognostic factors

**Fig. 1:**
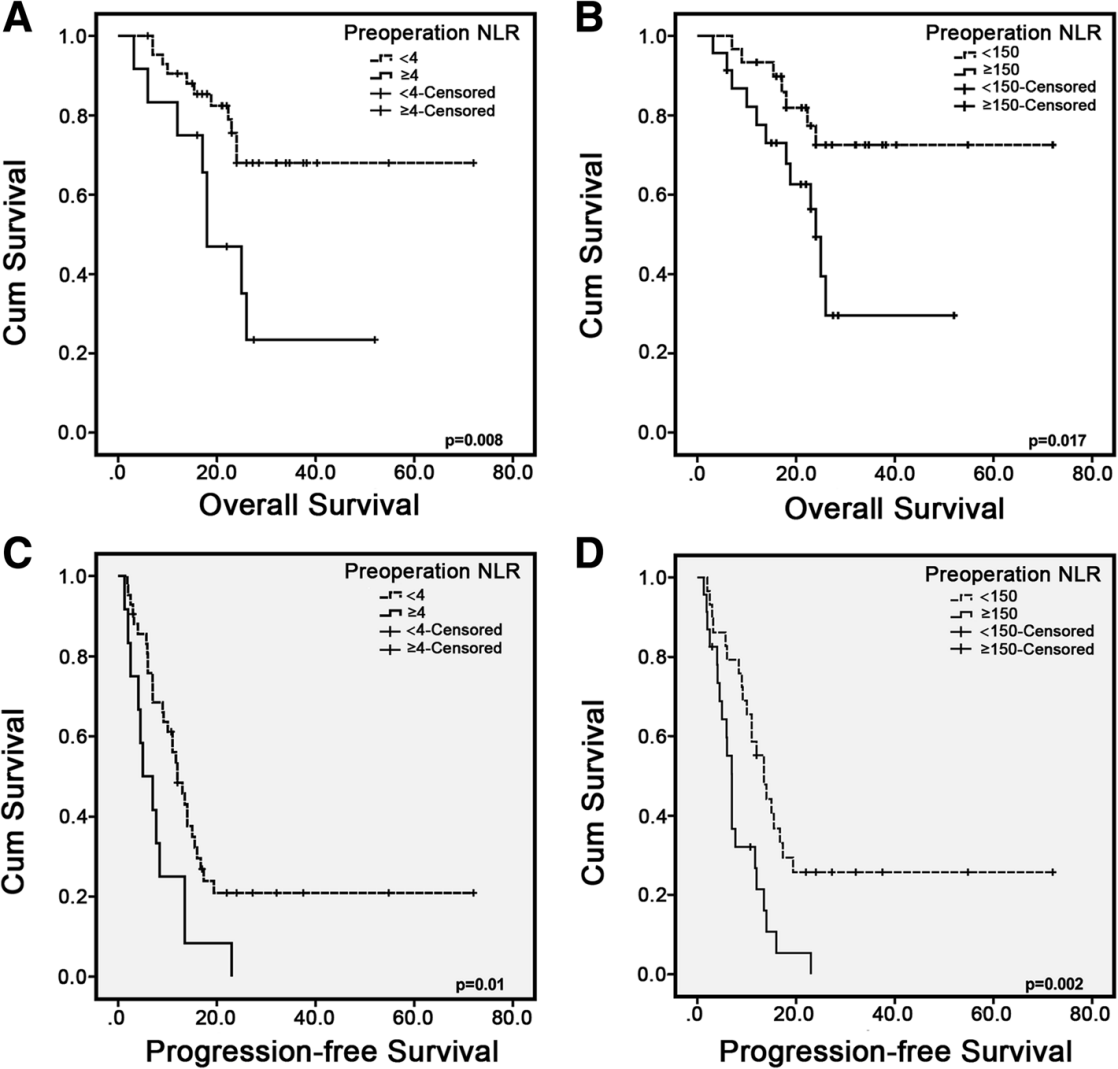
Patients with elevated NLR and PLR had worse prognosis. **a**, **b** Patients with higher NLR and PLR tended to have worse overall survival (*p* = 0.008 and *p* = 0.017). **c**, **d** Patients with higher NLR and PLR tended to have worse progression-free survival (*p* = 0.01 and *p* = 0.002)

Median OS was significantly longer in patients with NLR < 4 and PLR < 150, which was 24 to 56 months in the NLR groups (*p* = 0.008) and 27 to 56 months in the PLR groups (*p* = 0.017) (Fig. 1a, b). Results of median PFS coincided with those of OS, which was 7 to 23 months in the NLR groups (*p* = 0.01) and 8 to 26 months in the PLR groups (*p* = 0.002) (Fig. 1c, d).

Tables 3 and 4 show the results of univariate and multivariate analyses of various parameters evaluated in this study. Univariate analysis revealed that number of metastases >3, no resection of liver metastases, and elevated NLR and PLR were significantly associated with worse OS. However, according to multivariate analysis, only resection of liver metastases was a significant independent prognostic factor (*p* = 0.002).

**Table 3:** Prognostic factors associated with OS and PFS in univariate analysis

Prognosis variables	Overall survival		Progression-free survival	
	*p* value	HR (95% CI)	*p* value	HR (95% CI)
Gender, male/female	0.986	1.009 (0.384–2.651)	0.579	1.197 (0.635–2.254)
Age, ≥60/<60	0.109	2.142 (0.844–5.440)	0.007*	0.397 (0.203–0.775)
Number of metastasis, ≥3/<3	0.049*	1.323 (1.011–1.809)	0.015*	2.167 (1.159–4.054)
Liver metastasis resection, yes/no	0.001*	0.028 (0.012–0.049)	0.005*	0.313 (0.138–0.710)
Chemotherapy, FOLFOX/XELOX	0.735	0.855 (0.346–2.113)	0.891	0.925 (0.522–1.759)
Maximum size (cm), ≤4/>4	0.261	2.385 (0.525–10.84)	0.620	1.164 (0.638–2.123)
Pathology, adenocarcinoma/mucinous	0.261	2.385 (0.525–10.84)	0.737	1.225 (0.376–3.993)
Differentiation, G1–G2/G3–G4	0.930	0.958 (0.371–2.478)	0.302	1.386 (0.745–2.580)
Location, colon/rectum	0.421	1.159 (0.809–1.659)	0.446	1.265 (0.691–2.314)
Tumor stage (T)				
T2/T3	0.835	6.231 (0.926–52.51)	0.054	7.826 (0.962–63.66)
T2/T4	0.554	1.339 (0.460–4.251)	0.094	2.410 (0.899–4.843)
Nodal status (N)				
N0/N1	0.496	0.637 (0.174–2.330)	0.811	0.908 (0.412–2.001)
N0/N2	0.759	0.859 (0.326–2.265)	0.396	0.396 (0.369–1.483)
Lymphovascular invasion, no/yes	0.399	0.683 (0.281–1.658)	0.867	0.950 (0.520–1.735)
Perineural invasion, no/yes	0.289	1.613 (0.667–3.899)	0.064	1.775 (0.967–30257)
Extranodal tumor deposits, no/yes	0.340	1.542 (0.633–3.755)	0.549	1.201 (0.660–2.186)
MSI status, pMMR/MMR	0.371	0.606 (0.202–1.817)	0.542	0.673 (0.189–2.404)
NLR, ≥4/<4	0.013*	3.182 (1.277–7.933)	0.017*	2.284 (1.156–4.498)
PLR, ≥150/<150	0.024*	2.954 (1.155–7.551)	0.002*	2.535 (1.339–4.779)
ALP, elevated/normal	0.905	0.927 (0.267–3.217)	0.374	1.449 (0.640–3.282)
LDH, elevated/normal	0.257	1.810 (0.650–5.041)	0.008	2.673 (1.292–5.528)
Serum CA199 level, elevated/normal	0.343	1.640 (0.590–4.560)	0.700	1.135 (0.596–2.158)
Serum CEA level, elevated/normal	0.487	1.552 (0.450–5.351)	0.669	1.183 (0.548–2.553)

*A *p* value ≤0.05 was considered statistically significant

**Table 4:** Prognostic factors associated with OS and PFS in multivariate analysis

Prognosis variables	Overall survival		Progression-free survival	
	*p* value	HR (95% CI)	*p* value	HR (95% CI)
Age, <60/≥60			0.018*	2.373 (1.162–4.847)
Number of metastasis, <3/≥3	0.781	0.843 (0.251–2.823)	0.582	1.309 (0.501–3.423)
Liver metastasis resection, yes/no	0.002*	0.003 (0.012–0.033)	0.017*	0.180 (0.058–0.554)
NLR, ≥4/<4	0.477	1.511 (0.212–2.064)	0.520	1.334 (0.312–1.801)
PLR, ≥150/<150	0.510	1.447 (0.231–2.070)	0.027*	2.591 (0.166–0.896)
LDH, normal/elevated			0.011*	0.310 (0.125–0.768)

*A *p* value ≤0.05 was considered statistically significant

With regard to PFS, the parameters mentioned above, together with elevated LDH level and patient age <60 years, correlated with worse PFS. Multivariate analysis revealed that age (*p* = 0.018), resection of liver metastases (*p* = 0.017), LDH level (*p* = 0.011), and PLR (*p* = 0.027) were significant independent prognostic factors.

### Normalization of NLR and PLR before cycle 2 and correlations with PFS and OS

Patients were categorized into three groups as follows: group 1, patients with NLR < 4 or PLR < 150 at baseline (*n* = 41 and 31, respectively); group 2, patients with NLR ≥ 4 or PLR ≥ 150 at baseline, which decreased before cycle 2 of chemotherapy (*n* = 8 and 11, respectively); and group 3, patients with a higher NLR or PLR, which did not decrease before cycle 2 of chemotherapy. As presented in Table 5, patients with normalization of NLR or PLR before cycle 2 of chemotherapy showed improved disease control (*p* = 0.038 and 0.025, respectively). However, no significant change in objective chemotherapy response was observed, which coincided with the results of preoperative NLR and PLR.

**Table 5:** Chemotherapy response rate according to the alteration in NLR and PLR before the second cycle of treatment

Response	NLR				PLR			
	Group 1 (*N* = 41)	Group 2 (*N* = 8)	Group 3 (*N* = 6)	*p* value	Group 1 (*N* = 31)	Group 2 (*N* = 11)	Group 3 (*N* = 13)	*p* value
Objective response rate (CR + PR), % (cases)	20% (8)	25% (2)	0 (0)	0.070	19% (6)	18% (2)	0 (0)	0.126
Disease control rate (CR + PR + SD), % (cases)	56% (23)	38% (3)	0 (0)	0.038*	61% (19)	45% (5)	15% (2)	0.025*

NLR: group 1, <4; group 2, ≥4 → <4; group 3, ≥4 → ≥4. PLR: group 1, <150; group 2, ≥150 → <150; group 3, ≥150 → ≥150

*A *p* value ≤0.05 was considered statistically significant. (group 2 compared with group 3)

**Fig. 2:**
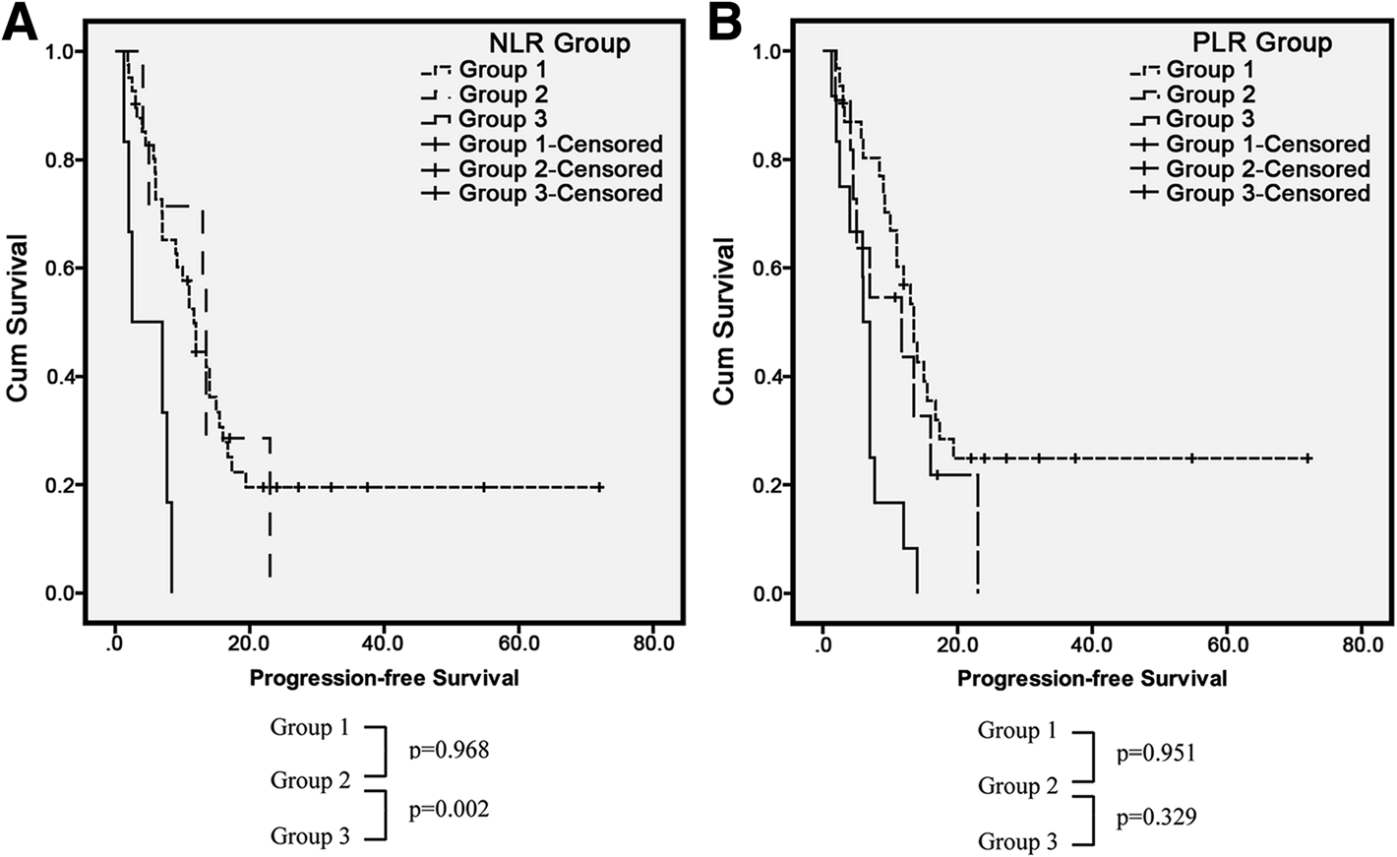
Changes in PFS with normalization of NLR and PLR. **a** Patients with normalization of NLR had better PFS (*p* = 0.002) than those with stable NLR levels. **b** PFS of patients with normalization of PLR did not differ (*p* = 0.329)

Finally, we conducted subgroup analysis according to changes in NLR and PLR between two stages of the treatment process (Fig. 2a, b). Patients with normalization of NLR had significantly better PFS than those with a high NLR that did not decrease (*p* = 0.002). However, although a tendency of better PFS was detected in patients with reduced PLR, the difference was not significant (*p* = 0.329). Moreover, there was no significant difference in OS (data not shown).

## Discussion

To our knowledge, this is the first study to evaluate the associations of NLR and PLR with prognosis and chemotherapy response in patients with synchronous CLM who underwent palliative resection of the primary tumor followed by oxaliplatin-based chemotherapy. Our results support the use of NLR and PLR as markers to predict chemotherapy response and prognosis, which would help to evaluate the possibility of secondary surgery for CLM as well as to elucidate the survival rates in such patients.

Immune cells act closely with tumor development, while NLR reflects systematic inflammation. Several studies have revealed the prognostic role of NLR in patients with CRC [6]. Although its mechanism has not been clarified, several investigations have deduced its close correlations with interleukin 6, interleukin 8, vascular epidermal growth factor, and other cytokines, which play important roles in tumorigenesis [18–20]. However, most studies focused on the use of NLR as a predictor in advanced CRC. Vauthey first reported that a high NLR independently predicted worse OS in patients with CLM treated with chemotherapy followed by hepatic resection or chemotherapy alone [21]. Other studies focusing on unresectable CLM have achieved similar results indicating NLR as an independent predictor of survival [22, 23]. In our study, we confirmed that a high NLR predicted worse OS and PFS. However, we did not achieve the same result in multivariate analysis, which somewhat conflicts with the studies mentioned above. A possible reason might be the difference in the study group we enrolled, which was confined to synchronous CLM with palliative resection of the primary lesion. However, the close correlation between NLR and chemotherapy response, i.e., the possibility of metastasis resection, might cover the statistical difference in the multivariate analysis model.

The mechanism of PLR in tumorigenesis might be derived from the role of platelets in promoting angiogenesis, adhesion, and invasion by increasing the production of vascular epidermal growth factor and transforming growth factor-beta in a tumor environment [24]. Meanwhile, cytokines and chemokines released from platelets facilitate other immune cells, including neutrophils and lymphocytes, to infiltrate into tumor lesions and trigger inflammatory progress [25]. Contrary to the wide study of NLR in CRC, fewer studies have focused on PLR. Xia demonstrated that in metastatic CRC, NLR was superior to PLR for predicting prognosis; in fact, NLR was the only independent predictor [26]. This finding was confirmed by another study in which preoperative NLR and derived NLR, but not PLR, were associated with worse OS and cancer-specific survival [27]. However, in patients with liver-only colorectal metastases and hepatectomy, Mudan found the reverse result, in which the prognostic effect of preoperative PLR was superior to that of NLR [28]. In our study, we also found that patients with a high preoperative PLR had worse PFS and OS and that only PLR was an independent predictor of PFS. However, its impact on OS was not observed, which might also be due to the interaction with metastasis resection. Overall, we demonstrated a better prognostic value of PLR than NLR.

NLR and PLR also have been reported as markers of chemotherapy response. In Clarke’s research, they enrolled two independent cohorts with unresectable CRC who received first-line palliative chemotherapy and found that normalization of NLR after one cycle of chemotherapy resulted in improved PFS [24]. Meanwhile, in a study of patients with advanced or recurrent unresectable CRC who received oxaliplatin-based combination chemotherapy, Kitayama also found better disease control in those with a low NLR [25]. Our results are in agreement with these previous studies showing that patients with a low NLR benefit more from first-line oxaliplatin-based chemotherapy. Patients with a dramatic decrease in NLR after one cycle of chemotherapy also demonstrated improved disease control and PFS, as its reduction might reflect the sensitivity to chemotherapy. Moreover, we found the same results with regard to PLR. However, we only found a tendency of improved PFS in those with normalization of PLR, which might be due to the limited number of patients in our study.

The current study had several limitations that are common in retrospective investigations. With an increased sample size and expanded follow-up, the impact of NLR and PLR in such patients would be ascertained. In addition, we did not evaluate the adverse reactions from chemotherapy, which might have affected the patients’ quality of life and survival. Finally, we did not consider or standardize the subsequent therapeutic strategy, which correlated closely with OS.

## Conclusions

In conclusion, our results suggest that preoperative NLR and PLR and their normalization might be good markers for better disease control in patients with synchronous CLM. PLR was better than NLR for predicting PFS in this study. Based on these findings, we have a better understanding of these patients, which may help to guide their therapeutic strategy.

## Additional file

Additional file 1: X-tile plots of NLR and PLR to OS and PFS, respectively. *X*-axis represents all cutoff values applied from low to high (left to right) to define the subsets. Brighter pixels indicate a stronger association between markers and prognosis. Cutoff values were defined in the brightest pixels (marked by the bold black dots). A and B. Cutoff values of NLR were 3.9 to PFS and 3.7 to OS, respectively. C. Cutoff value of PLR to PFS ranged from about 135 to 220. D. Cutoff value of PLR to OS was 150.1. (TIF 3184.64 kb)

## Abbreviations

ALP: Alkaline phosphatase
CLM: Colorectal liver metastasis
CR: Complete response
CRC: Colorectal cancer
ECOG: Eastern Cooperative Oncology Group
LDH: Lactic dehydrogenase
mFOLFOX6: Folic acid/fluorouracil plus oxaliplatin
NLR: Neutrophil-to-lymphocyte ratio
OS: Overall survival
PFS: Progression-free survival
PLR: Platelet-to-lymphocyte ratio
PR: Partial response
SD: Stable disease
XELOX: Capecitabine plus oxaliplatin
