# Supplementary figures and images for: Neutrophil-to-lymphocyte and platelet-to-lymphocyte ratios predict chemotherapy outcomes and prognosis in patients with colorectal cancer and synchronous liver metastasis
Source: World J Surg Oncol. 2016 Nov 16;14:289. doi: 10.1186/s12957-016-1044-9 (PMC5112720; doi:10.1186/s12957-016-1044-9)

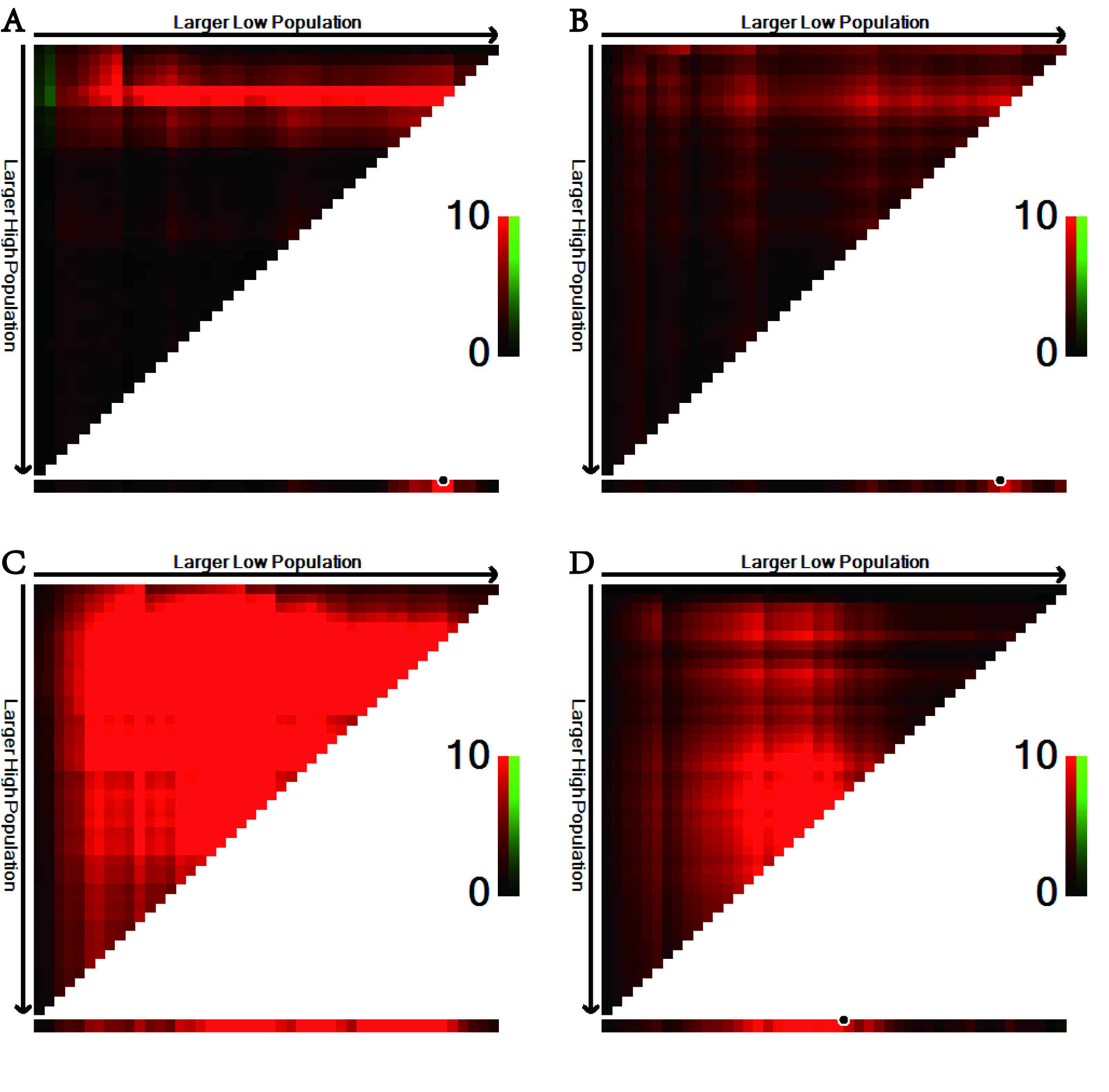

Supplement: Additional file 1: — X-tile plots of NLR and PLR to OS and PFS, respectively. X-axis represents all cutoff values applied from low to high (left to right) to define the subsets. Brighter pixels indicate a stronger association between markers and prognosis. Cutoff values were defined in the brightest pixels (marked by the bold black dots). A and B. Cutoff values of NLR were 3.9 to PFS and 3.7 to OS, respectively. C. Cutoff value of PLR to PFS ranged from about 135 to 220. D. Cutoff value of PLR to OS was 150.1. (TIF 3184.64 kb) [file 12957_2016_1044_MOESM1_ESM.tif]
